# Draft Genome Sequence of “*Candidatus* Synechococcus spongiarum” m9, Binned from a Metagenome of South China Sea Sponge *Theonella swinhoei*

**DOI:** 10.1128/genomeA.01307-16

**Published:** 2017-02-09

**Authors:** Fang Liu, Jinlong Li, Zhiyong Li

**Affiliations:** Marine Biotechnology Laboratory, State Key Laboratory of Microbial Metabolism and School of Life Sciences and Biotechnology, Shanghai Jiao Tong University, Shanghai, People’s Republic of China

## Abstract

“*Candidatus* Synechococcus spongiarum” represents the widespread cyanobacterial symbionts found in marine sponges with relatively high genomic variability and likely important ecological roles. We present here the draft genome sequence of “*Candidatus* Synechococcus spongiarum” m9, which was assembled from a metagenome of *Theonella swinhoei* sampled in the South China Sea.

## GENOME ANNOUNCEMENT

Members of “*Candidatus* Synechococcus spongiarum” are widespread in sponge symbiotic microbial communities (1). These cyanobacterial symbionts are uncultivated and may play important roles in primary production, UV protection, and chemical defense (2, 3). Application of metagenome binning and single-cell sequencing has revealed four “*Ca.* Synechococcus spongiarum” draft genomes from the sponges sampled in the Red Sea and the Mediterranean Sea (4, 5). Nonetheless, these four genomes varied in genome size, gene content, and immune system features, although their 16S rRNA gene sequences were highly similar (~99%) (5). Hence, it is necessary to obtain more genomes from different locations/hosts for the better understanding of the genomic diversity and adaptation mechanisms of “*Ca.* Synechococcus spongiarum.” None of the available “*Ca.* Synechococcus spongiarum” genomes are from the Pacific Ocean. Here, we describe the “*Ca.* Synechococcus spongiarum” m9 draft genome sequence recovered from the metagenome of a South China Sea sponge, *Theonella swinhoei*.

*Theonella swinhoei* was collected near Yongxing Island (112°20′E, 16°50′N) in the South China Sea. DNA extraction, Illumina sequencing, and metagenome assembling were carried out as described by Liu et al. (6). VizBin was used to extract the metagenomic bins from the metagenome contigs, using default settings (7). Contigs shorter than 2,000 bp were removed from each bin. PhyloSift (8) and JSpeciesWS (9) were used to determine the phylogenetic affiliation of each bin. The completeness, contamination rate, and average amino acid index (AAI) were calculated by enveomics-GUI (https://github.com/lmrodriguezr/enveomics-gui), according to Liu et al. (6). The RASTtk pipeline (10) and PATRIC (11) were used for genome annotation and comparative analysis. Additionally, WebMGA (12) was used for COG annotation.

The above-described process resulted in a cyanobacterial metagenome bin, namely, “*Ca.* Synechococcus spongiarum” m9. This genome consists of 179 contigs with a genome size of 1.4 Mb (*N*_50_, 10.7 kb) and a G+C content of 61.9%. enveomics-GUI indicated a genome completeness of 93%, with likely no contamination. Phylogenetically, it is closely related to “*Ca.* Synechococcus spongiarum” SP3 from the *T. swinhoei* sampled from the Red Sea (5), as suggested by a higher AAI (86.87%) than the AAI values between other public “*Ca.* Synechococcus spongiarum” draft genomes (approx. 84 to 85%). The m9 genome contains 1,659 coding sequences (CDSs) (585 hypothetical proteins) and 33 tRNAs. It is smaller than any known “*Ca.* Synechococcus spongiarum” genome (5). This cyanobacterial symbiont has a metabolism repertoire similar to that of the other “*Ca.* Synechococcus spongiarum” strains, based on PATRIC analysis. Seventy-nine percent of the previously defined “*Ca.* Synechococcus spongiarum” “core COGs” (5)” exist in the m9 genome. Like its relatives, the m9 genome is short of antioxidant enzymes and small peptides of photosystem II, which are regarded to be adaptive traits to the symbiotic lifestyle (5). Nonetheless, genes of biosynthesis of dTDP-l-rhamnose (O antigen), the absence of which was regarded to be a signature of “*Ca.* Synechococcus spongiarum” (5), were found in m9. This observation underlines the variability of “*Ca.* Synechococcus spongiarum” genomes and suggests the diverse mechanisms in sponge-cyanobacterium recognition.

### Accession number(s).

The “*Ca.* Synechococcus spongiarum” m9 draft genome has been deposited at ENA with the contig accession range FITM01000001 to FITM01000179.
